# Recent Trends in Prostate Biopsy Complication Rates and the Role of Aztreonam in Periprocedural Antimicrobial Prophylaxis—A Nationwide Population-Based Study from Korea

**DOI:** 10.3390/antibiotics11030312

**Published:** 2022-02-25

**Authors:** Wook Nam, Min Uk Park, Han Kyu Chae, Jihye Song, Han Gwun Kim, Jong Yeon Park, Seokjoon Lee, Sung Jin Kim

**Affiliations:** 1 Department of Urology, Gangneung Asan Hospital, University of Ulsan College of Medicine, Gangneung 25440, Korea; wooki6258@gnah.co.kr (W.N.); sinopoli@gnah.co.kr (H.K.C.); hgkim@gnah.co.kr (H.G.K.); jypark@gnah.co.kr (J.Y.P.); 2 Department of Urology, Asan Medical Center, University of Ulsan College of Medicine, Seoul 05505, Korea; dnrl1112@gmail.com; 3 Department of Neurosurgery, Ajou College of Medicine, Ajou University Hospital, Suwon 16499, Korea; 4 Department of Pharmacology, Catholic Kwandong University College of Medicine, Gangneung 25601, Korea; sjlee@kwandong.ac.kr

**Keywords:** aztreonam, prostate biopsy, prophylactic antibiotic, susceptibility

## Abstract

An increase in the rate of complications after prostate biopsy (PB) due to increased antibiotic-resistant bacteria is a global issue. We report the safety of aztreonam as a prophylactic antibiotic in patients undergoing PB. We investigated the complication rates according to several antibiotic regimens, including aztreonam. We hypothesized that PB complications increased following a rise in antibiotic-resistant bacteria. We examined the annual rates of complications among patients in our hospital (clinical cohort) and the Korea Health Insurance Review and Assessment Service (HIRA) cohort. Data regarding complications, hospitalization, emergency room (ER) visits, and febrile urinary tract infections occurring within 2 weeks after PB were recorded. The rate of complications was significantly lower in patients who received oral quinolone and intravenous aztreonam than in those who received oral quinolone. The complication rates did not increase throughout the study period. Additionally, 1754 patients from the HIRA cohort were included. The rates of complications, hospitalizations, and ER visits did not increase among these patients. Oral quinolone combined with intravenous aztreonam reduced the rate of febrile complications compared to quinolone alone and was safe to use after PB. Therefore, we recommend intravenous aztreonam with oral quinolone as a prophylactic antibiotic regimen before PB.

## 1. Introduction

Prostate cancer is the fifth most common cancer and has the second-fastest increase in prevalence in men, according to the Korean National Statistical Centre [1]. Transrectal ultrasonography-guided prostate biopsy (PB) is the gold standard method for collecting prostate tissue to diagnose prostate cancer. Although PB is a safe procedure performed in an outpatient setting, post-procedural complications include urinary tract infection, hematuria, hemospermia, and urinary obstruction. Although rare, uncontrolled febrile urinary tract infection may progress to sepsis as a severe condition [2]. The number of patients requiring a PB is increasing worldwide, as the prostate-specific antigen (PSA) is being used as a standard to screen for prostate cancer. Although PB is a safe procedure, the number of patients requiring hospitalization due to complications has increased in several regions over the past 10 years. It can be correlated to the increase in the number of antibiotic-resistant bacteria [3,4]. In addition to increasing the frequency of infection-related complications by PB, 1,300,000 patients are diagnosed with prostate cancer annually worldwide, resulting in a high absolute number of complications post prostate biopsies [2,5].

Aztreonam is the only clinically used monobactam antibiotic in the β-lactam family [6]. This antimicrobial is effective against gram-negative bacteria, including *Escherichia coli*, the pathogen causing 75–90% of post-PB infection-related complications [7]. Additionally, it was expected to be effective for urinary tract infections because it maintains a high concentration in urine [8] and prostate tissue [9]. Nonetheless, there is no report on its large-scale use. Fluoroquinolone are widely used and recommended as prophylactic antibiotics because they reduce complications after PB since they are effective against gram-negative bacteria, including *E. coli* [7,10,11]. In the past, the frequency of urinary tract infections after a PB was 2–6%, and the risk of sepsis was 0.2–2% [7]. Nevertheless, readmission rates are increasing, with the most severe complications caused by resistant bacteria [12,13]. In addition, it is unclear which regimen of antibiotics is appropriate because the prevalence and complications of resistant bacteria differ by region [14,15].

We investigated whether complications of PB increase with time using data from the Korea Health Insurance Review and Assessment Service (HIRA) cohort and clinical data from PB performed at our institution. We also determined whether the rates of infection caused by resistant bacteria increased after prostate biopsies. Furthermore, the complication rates of several antibiotic regimens were compared using data from PB performed at our institution.

## 2. Results

**Figure 1:**
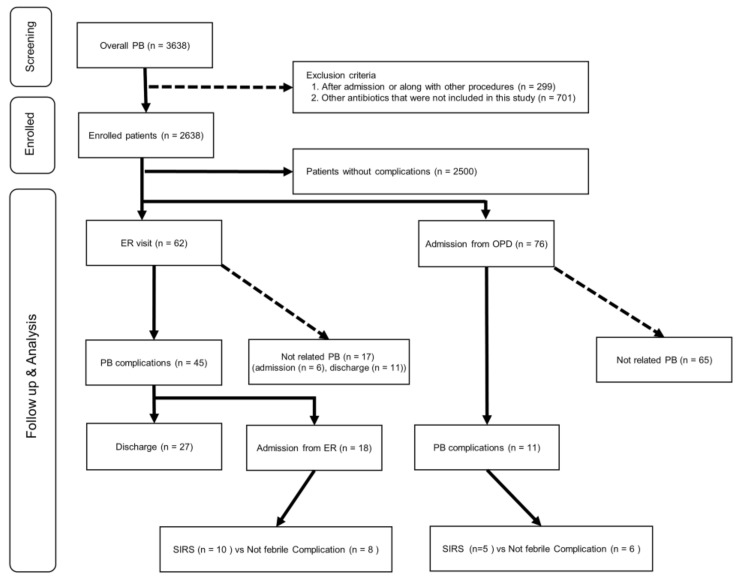
Patient flowchart for the clinical cohort. ER, emergency room; OPD, outpatient department; PB, transrectal-ultrasonography-guided prostate biopsy; SIRS, systemic inflammatory response syndrome.

The patient flowchart of the clinical dataset is shown in Figure 1. The clinical data of 3628 patients who underwent PB at our hospital from January 1997 to October 2019 were collected, and 1000 patients were excluded from this study. The final analysis included 2638 patients.

**Figure 2:**
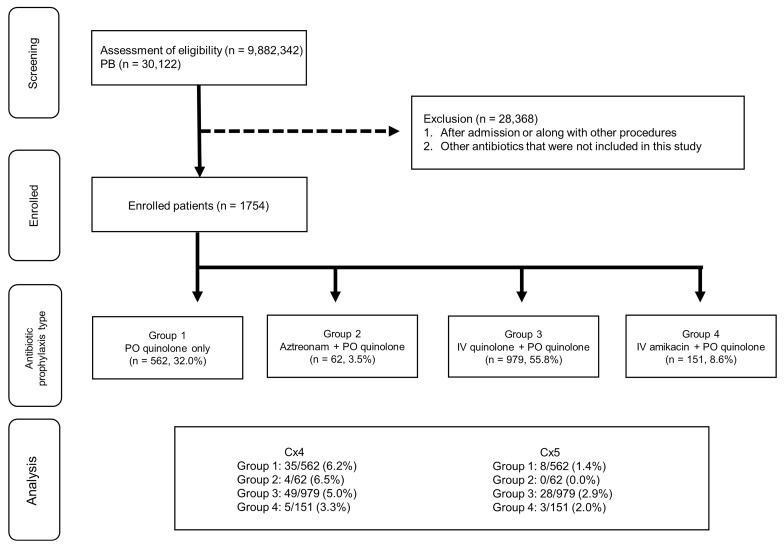
Patient flowchart for The Korea Health Insurance Review and Assessment Service (HIRA) cohort. Group 1: patients who were prescribed oral quinolone as a prophylactic antibiotic for PB; Group 2: patients who were prescribed oral quinolone and intravenous aztreonam as prophylactic antibiotics for PB; Group 3: patients who were prescribed oral quinolones and intravenous aminoglycosides as prophylactic antibiotics for PB; Group 4: patients who were prescribed oral quinolones and intravenous quinolones as prophylactic antibiotics for PB. Cx4: patients with a history of visiting the ER; Cx5: patients with a history of hospitalization. IV, intravenous; PO, per oral.

The patient flowchart for the HIRA cohort is shown in Figure 2. Data from 9,882,342 patients were analyzed, of which 30,122 patients underwent PB. The final analysis included 1754 patients after 28,368 patients were excluded. These patients were divided into four groups: Group 1 (*n* = 562), Group 2 (*n* = 62), Group 3 (*n* = 979), and Group 4 (*n* = 151).

Table 1 shows patient characteristics. The patients in Group 4 tended to be younger than those in the other groups; Group 3 had the highest number of biopsy cores, whereas Group 4 had the lowest number of biopsy cores (*p* < 0.01). The patients in groups 1 and 4 had lower rates of prostate cancer, as a pathologic result, than those in groups 2 and 3 (*p* < 0.01). The patients in Group 2 had a higher rate of hypertension than those in Group 1. There were no differences in PSA, body mass index, diabetes mellitus (DM), or pulmonary diseases among the groups (*p* < 0.01). There were no differences in the rates of patients who visited the emergency room (ER) or the outpatient department as a complication of PB (Cx1) and patients with a history of visiting the ER (Cx4) between the groups. Group 1 had a significantly higher rate of patients requiring hospitalization due to a complication of PB (Cx2), patients with a PB complication meeting the criteria for systemic inflammatory response syndrome (SIRS) (Cx3), and patients with a history of hospitalization (Cx5) than the other three groups (*p* < 0.01 all).

Table 2 shows the characteristics of the clinical cohort according to the year in which PB was performed. Patients who underwent PB in 2016–2017 were significantly older than those who underwent PB in 1997–2007, 2010–2011, 2012–2013, and 2014–2015 (all *p* < 0.01). The patients who underwent PB in 2018–2019 were significantly older than those who underwent PB in 1997–2007 (*p* < 0.01) and in 2012–2013 (both *p* = 0.01). The patients who underwent PB in 2008–2009 were significantly younger than those who underwent PB in 2016–2017, 2018–2019 (all *p* < 0.01). The mean PSA levels of patients who underwent PB in 1997–2007, 2008–2009 were significantly higher than those of the patients who underwent PB in 2012–2013, 2014–2015, 2016–2017, and 2018–2019 (all *p* < 0.01). The number of biopsy cores in patients who underwent PB increased significantly from 1997–2007 to 2016–2017 (*p* < 0.01). The prostate cancer detection rate in 1997–2007 was lower than in 2014–2015, 2016–2017, and 2018–2019 (all *p* < 0.01). There was no correlation between prostate cancer and year.

**Figure 3:**
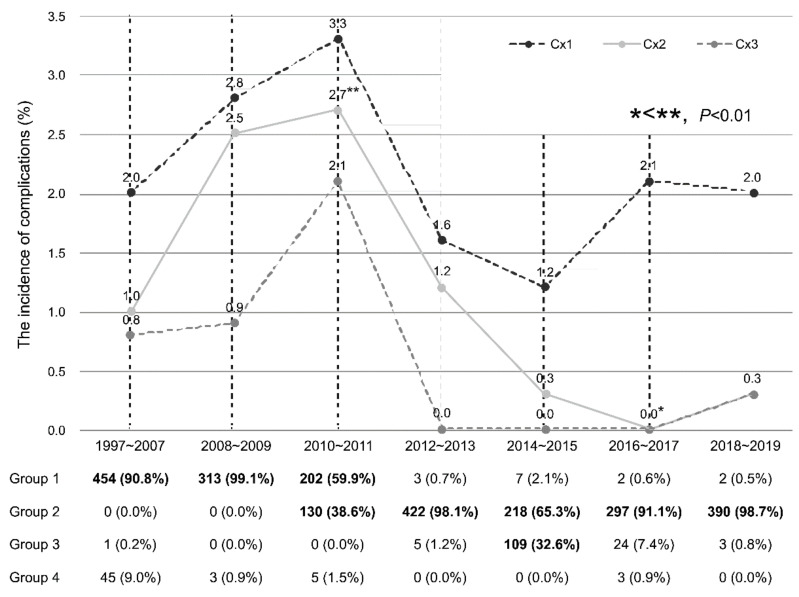
The incidence of complications after PB in the clinical cohort according to the year. Groups accounting for 30% or more are displayed in bold. Cx1: patients who visited the ER or outpatient department as a complication of PB; Cx2: patients requiring hospitalization due to a complication of PB; Cx3: patients with a PB complication meeting the criteria for SIRS. Group 1: patients who were prescribed oral quinolone as a prophylactic antibiotic for PB; Group 2: patients who were prescribed oral quinolone and intravenous aztreonam as prophylactic antibiotics for PB; Group 3: patients who were prescribed oral quinolones and intravenous aminoglycosides as prophylactic antibiotics for PB; Group 4: patients who were prescribed oral quinolones and intravenous quinolones as prophylactic antibiotics for PB.

Figure 3 shows the incidence of complications and regimen of prophylactic antibiotics after PB in the clinical cohort according to the year. Most patients were prescribed the prophylactic antibiotic of Group 1 in 1997–2007, 2008–2009, and Group 2 in 2012–2013, 2016–2017, and 2018–2019. Group 1 and Group 2 regimens were used in 59.9% of patients in 2010–2011 and 65.3% in 2014–2015, respectively. Additionally, the majority of the remaining patients were prescribed Group 2 antibiotics in 2010–2011 and Group 3 regimens in 2014–2015. There was no recent increase in complications of Cx1, Cx2 and Cx3 in each period. Cx2 occurred more frequently in 2010–2011 than in 2016–2017 (*p* < 0.01)

**Figure 4:**
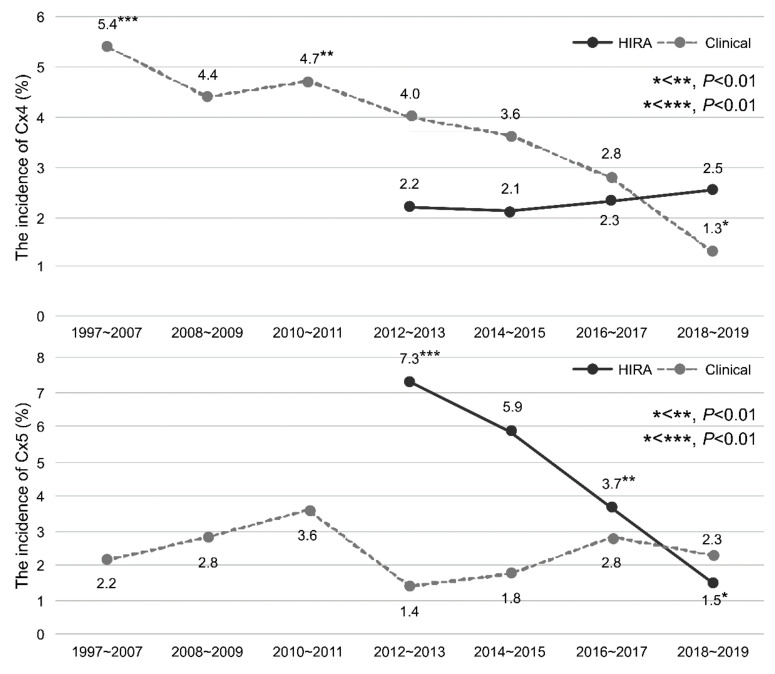
The incidence of complications after PB according to the year in the HIRA and clinical cohorts. PB: transrectal-ultrasonography-guided PB.

The rates of Cx4 and Cx5 in the clinical and HIRA cohorts are shown by year and group in Table 3 and Figure 4. The rate of Cx4 was significantly lower in the clinical cohort in 2018–2019 than in 1997–2007 and 2010–2011 (*p* < 0.01). The rate of Cx5 did not differ by year in the clinical cohort. The rate of Cx5 in the HIRA cohort was lower in 2018–2019 than in 2012–2013 and 2016–2017 (all *p* < 0.01). There was no significant difference in the rate of Cx4 expression in the HIRA cohort by year.

The rate of Cx3 expression was significantly lower in Group 2 than in Group 1 (*p* = 0.004) (Table 4). No significant risk factors for Cx3 were found; therefore, a multivariate analysis was not performed. The bacteria isolated from the urine or blood of Cx3 and their susceptibility to antibiotics are shown in Table 5. Two patients had extended-spectrum beta-lactamases (ESBL) in both urine and blood in 2010 and 2019. Two patients were found to have quinolone-resistant *E. coli* in the blood; one patient underwent PB biopsy in 2010 and the other in 2019. Therefore, the number of patients from which antibiotic-resistant bacteria were cultured in urine or blood was low.

## 3. Discussion

Oral quinolone with IV aztreonam reduced the complications of febrile urinary tract infections more effectively than oral quinolone alone. In recent years, there has been no increase in post-PB complications in patients prescribed a prophylactic antibiotic regimen after PB.

The incidence of infectious complications was reported to increase over time after PB [2,3,16]. Loeb et al. [2] reported that 67 patients (0.38%) required hospitalization for infectious complications following PB, with an increasing trend from 0.4% in 1991 to 1.1% in 2007. Nam et al. [3] reported that the number of patients who needed hospitalization increased four-fold to 4.1% in 2005 compared to 1996. The main reason for this was believed to be the increase in infectious complications. The European Association of Urology (EAU) group conducted a prospective study on complications after PB and found that 5.2% of patients had symptomatic urinary tract infections, and 3.5% developed urinary tract infections accompanied by fever. Furthermore, the increases in complications may be attributed to an increase in fluoroquinolone-resistant bacteria [16]. Figure 4 shows the incidence of complications over time in HIRA and clinical cohorts. The incidence of infectious complications requiring hospitalization (Cx3) and other complications requiring hospitalization (Cx2) was lower than that in previous reports, and the incidence of complications after PB did not increase over the study period

An increase in the resistance of *E. coli* to fluoroquinolones has been reported in the United States [17] and worldwide [18,19]. The European Centre for Disease Control regularly reports antibiotic resistant strains in specific geographical regions. In the 2018 report, approximately half of the European nations reported that 10–25% of *E. coli* strains were antibiotic resistant. The remaining European countries reported that 25–50% of *E. coli* were resistant to fluoroquinolones, with an overall estimated population-weighted mean value of 25.3%, which is increasing over time [20]. Another study reported that fluoroquinolone-resistant *E. coli* increased from 21.5% in 2007 to 25.4% in 2016 [19]. The increase in fluoroquinolone-resistant *E. coli* is related to exposure to broad-spectrum antibiotics [20] and the prevalence of high-resistance *E. coli* in food-grade animals [21]. It is estimated that the close geographical regions and active trade practices of the European Union contribute to an increased incidence of fluoroquinolone-resistant *E. coli* via the exchange of contaminated meat [20,21].

A Korean study conducted between 2005 and 2012 on 5577 patients who underwent PB reported that 0.48% of the total study group reported febrile urinary tract infections as a complication; however, the rate of complications did not increase over time [22]. A Japanese study conducted between 2004 and 2006 included 212,065 patients who underwent PB and found a rate of febrile complications of 1.1% and a rate of complications requiring hospitalization of 0.69% [23]. Therefore, the febrile urinary tract infection (Cx3) complication rate noted in this study (0.56%) and the rate of Cx2 (1.09%) were similar to the results reported by previous studies conducted in a relatively close geographical area.

Aztreonam is the only monobactam antibiotic used clinically in the β-lactam family. It was approved by the US Food and Drug Administration and the European regulatory authorities in 1986 [6]. It is effective against gram-negative bacteria that cause urinary tract infections, with a high concentration reported in urine [8] and prostate tissue [9]. However, in common gram-negative resistant bacteria such as ESBL, chromosomally encoded AmpC β-lactamases express more than one type of β-lactamase concurrently. Since Aztreonam is not stable when more than one β-lactamase is expressed simultaneously, multidrug-resistant (MDR) bacteria develop resistance through this mechanism [24]. The EAU recommends using fluoroquinolones as prophylactic antibiotics due to their high bioavailability and high concentration in prostate tissue [11]. However, the most important factor in selecting an antibiotic regimen is the fluoroquinolone resistance pattern, which increases with time in some regions. The incidence of resistant bacteria is also affected by the geographical region; therefore, it is necessary to find an appropriate antibiotic in the region over time [14,15].

In 2015, a systematic review reported that targeted antimicrobial prophylaxis helped reduce infective complications after PB based on bacteria cultured from rectal swabs [25]. However, this systematic review was limited by the fact that more than half of the included studies were conducted in the United States, which has a high incidence of resistant bacteria. A similar study conducted in the UK reported no difference in the rate of febrile urinary infections between patients treated with antibiotics deemed to be effective via a rectal swab and fluoroquinolones. These conflicting results among previous studies may be due to differences in the study populations [26]. Generally, the risk factors associated with PB complications include age, comorbidities, prostate enlargement, travel history, and recent use of fluoroquinolones [26,27]. Samarinas et al. [28] reported that administration of meropenem instead of ciprofloxacin concomitantly with amoxicillin/clavulanate reduces the rate of infectious complications in high-risk patients. However, the adverse effects on bacterial resistance pattern changes should be considered after exposing a large population to meropenem. The use of all four antibiotic regimens included in this study resulted in no increase in the rate of complications over time after PB.

To the best of our knowledge, this is the largest population-based clinical report on the use of aztreonam as a prophylactic antibiotic in patients undergoing PB (*n* = 1457). The pharmacokinetic and pharmacodynamic properties of aztreonam have recently been reviewed, as it is a potential antibiotic to treat the global spread of MDR gram-negative bacteria [29]. This study found that aztreonam is safe for prophylactic use in patients undergoing PB.

However, this study has some limitations. First, under-reporting is a potential issue considering the study’s retrospective nature and one-center design, since those treated for complications at a nearby hospital were not included as patients with complications. Nonetheless, we can assume that most patients with complications after PB revisited our hospital, since there is no other hospital within 100 km of our institution that can manage PB-related complications. As Korea is a country where everyone has health insurance, data from the HIRA cohort could be used in this study. However, with only this billing data, the exact reason for hospitalization or ER visits is often unclear. Therefore, we included the Cx4 and Cx5 outcomes to incorporate all the reasons for hospitalization and ER visits. This improved the accuracy of our results by validating the complication incidence for each study year.

## 4. Materials and Methods

### 4.1. Patient Eligibility and Study Design

This study consisted of two datasets: clinical data obtained from patients who underwent PB at our institution and data from patients in the HIRA cohort. Clinical data were obtained from 3638 patients who underwent PB at our hospital between 1997 and 2019. Patients who underwent PB simultaneously with other procedures or surgery and those who underwent PB while hospitalized were excluded (Figure 1). We collected data regarding age, serum PSA levels, number of biopsy cores, prostate cancer diagnoses, DM, hypertension, pulmonary diseases, ER visit history, hospitalization history, and clinical information related to hospitalization (laboratory values, vital signs, and detailed information obtained in the outpatient department or ER).

The HIRA dataset contains data regarding patient hospitalizations, outpatient visits, and ER visits. The data of patients who underwent PB between 2012 and 2018 were screened using the ICD-10 code. Patients who underwent PB and were prescribed oral quinolone as a discharge drug (on post-procedure days 1–7) were included in the study. Patients were divided into four groups according to the types of antibiotics prescribed: oral quinolone only (Group 1), oral quinolone and IV aztreonam (Group 2), oral quinolone and IV aminoglycosides (Group 3), and oral and IV quinolone (Group 4). Patients who were prescribed other antibiotics were excluded from the study.

### 4.2. Outcomes

To analyze the incidence of complications according to the type of antibiotic prescribed, patients who experienced procedure-related complications within 2 weeks of the PB and were treated in the ER or an outpatient setting were categorized as Cx1. Patients who required inpatient treatment for procedure-related complications within 2 weeks of PB were categorized as Cx2. Patients who required inpatient treatment due to SIRS within 2 weeks of PB were categorized as Cx3. Patients were diagnosed with SIRS when they met two or more of the following criteria: fever > 38.0 °C or hypothermia < 36.0 °C, tachycardia > 90 beats/min, tachypnea > 20 breaths/minute, and leukocytosis > 12 × 10^9^/L or leukocytopenia < 4 × 10^9^/L. Patients with a history of ER visits were categorized as Cx4, and those with a history of hospitalization were categorized as Cx5.

The increase in the number of patients requiring ER visits or inpatient care due to procedure-related complications after PB led us to hypothesize that the frequency of antibiotic-resistant bacteria has increased over time in Korea. We used clinical data (Cx1, Cx2, and Cx3) and additional patient data (Cx4 and Cx5) to determine the change in the complication rate over time.

### 4.3. Statistical Methods

Categorical variables were analyzed using Pearson’s chi-square test or Fisher’s exact test. Continuous variables were analyzed via analysis of variance or Kruskal–Wallis’ tests after checking the normality of distribution. When the null hypothesis was not rejected, a post-hoc test was performed using the Tukey method. Categorical variables are presented as frequency (percentage), whereas continuous variables are presented as medians (interquartile range). Univariate and multivariate logistic regression models were used to evaluate the effect of each variable on the rate of febrile urinary tract infection (Cx3). All statistical analyses were performed using SPSS (version 20.0; IBM Corporation, Armonk, NY, USA). Statistical significance was set at a two-tailed *p* value < 0.05.

## 5. Conclusions

Complications after PB due to an increase in antibiotic-resistant bacteria are a global problem; however, there is no evidence of such an increase in complications in Korea. The incidence of antibiotic-resistant bacteria differs according to geographic area. Oral quinolone combined with IV aztreonam was more effective in reducing the rate of febrile complications after PB than quinolone alone. This is a safe prophylactic antibiotic regimen for use after PB in Korea.

## Figures and Tables

**Table 1:** Baseline characteristics and complications of patients in the clinical cohort.

	Group 1 (*n* = 983)	Group 2 (*n* = 1457)	Group 3 (*n* = 142)	Group 4 (*n* = 56)	*p* Value
Age (median [IQR])	68.0 [62.0–73.0]	69.0 [63.0–74.0]	68.0 [62.0–73.0]	62.5 [57.0–69.0]	<0.001 *
PSA (median [IQR])	6.9 [5.3–11.6]	5.6 [4.1–8.4]	6.0 [3.8–8.0]	8.2 [5.5–14.0]	0.298 **
Cores (median [IQR])	12.0 [12.0–12.0]	12.0 [12.0–12.0]	14.0 [14.0–14.0]	2.0 [2.0–2.5]	<0.001 **
Prostate cancer (n)	339 (34.5%)	624 (42.8%)	60 (42.3%)	16 (28.6%)	<0.001 ***
BMI (median [IQR])	24.2 [22.0–25.0]	24.5 [22.9–26.5]	24.3 [22.7–26.3]	23.7 [22.4–25.0]	0.803 *
DM (n)	48/348 (13.8%)	247/1220 (20.2%)	19/141 (13.5%)	7/9 (22.2%)	0.059 ***
Hypertension (n)	147/347 (42.4%)	572/1229 (53.3%)	70/141 (49.6%)	6/9 (66.7%)	0.023 ***
Pulmonary disease (n)	12/329 (3.6%)	67/1186 (5.6%)	12/139 (8.6%)	0/8 (0.0%)	0.149 ***
Cx1 (n)	29 (3.0%)	24 (1.6%)	2 (1.4%)	1 (1.8%)	0.162 ***
Cx2 (n)	21 (2.1%)	7 (0.5%)	0 (0.0%)	1 (1.8%)	<0.001 ***
Cx3 (n)	12 (1.2%)	2 (0.1%)	0 (0.0%)	1 (1.8%)	0.002 ***
Cx4 (n)	28 (2.8%)	29 (2.0%)	4 (2.8%)	1 (1.8%)	0.552 ***
Cx5 (n)	59 (6.0%)	31 (2.1%)	3 (2.1%)	7 (12.5%)	<0.001 ***

* Analysis of variance, Tukey’s test. ** Kruskal–Wallis’ test, Tukey’s test. *** Pearson’s χ^2^ test or Fisher’s exact test, Tukey’s test. Group 1: patients who were prescribed oral quinolone as a prophylactic antibiotic for prostate biopsy (PB); Group 2: patients who were prescribed oral quinolone and intravenous aztreonam as prophylactic antibiotics for PB; Group 3: patients who were prescribed oral quinolones and intravenous aminoglycosides as prophylactic antibiotics for PB; Group 4: patients who were prescribed oral quinolones and intravenous quinolones as prophylactic antibiotics for PB. Cx1: patients who visited the emergency room (ER) or outpatient department as a complication of PB; Cx2: patients requiring hospitalization due to a complication of PB; Cx3: patients with a PB complication meeting the criteria for systemic inflammatory response syndrome (SIRS); Cx4: patients with a history of visiting the ER; Cx5: patients with a history of hospitalization. IQR, interquartile range; PSA, prostate-specific antigen; BMI, body mass index; DM, diabetes mellitus.

**Table 2:** Baseline characteristics and complications of patients in the clinical cohort by year.

Period	1997–2007 (*n* = 500)	2008–2009 (*n* = 316)	2010–2011 (*n* = 337)	2012–2013 (*n* = 430)	2014–2015 (*n* = 334)	2016–2017 (*n* = 326)	2018–2019 (*n* = 395)	*p* Value
Age (median [IQR])	67.0 [61.0–72.0]	67.0 [61.0–72.0]	68.0 [62.0–73.0]	68.0 [62.0–72.0]	68.0 [62.0–73.0]	71.0 [65.0–76.0]	69.0 [63.0–75.0]	<0.001 *
PSA (median [IQR])	7.4 [5.2–13.0]	7.0 [5.6–12.0]	6.4 [5.0–9.6]	5.8 [4.3–8.2]	5.6 [3.7–7.3]	5.5 [4.3–8.0]	5.3 [4.1–8.4]	<0.001 **
Cores (median [IQR])	10.5 [2.0–12.0]	12.0 [12.0–12.0]	12.0 [12.0–12.0]	12.0 [12.0–12.0]	13.0 [12.0–14.0]	14.0 [12.0–14.0]	12.0 [12.0–12.0]	<0.001 **
Prostate cancer (n)	159 (31.8%)	111 (35.1%)	125 (37.1%)	171 (39.8%)	148 (44.3%)	151 (46.3%)	174 (44.1%)	<0.001
BMI (median [IQR])	-	-	-	25.1 [23.5–27.1]	24.4 [23.0–26.2]	24.4 [22.6–26.4]	25.6 [21.7–27.7]	0.669 *
DM (n)	16/92 (17.4%)	15/130 (11.5%)	32/205 (15.6%)	53/308 (17.2%)	58/307 (18.9%)	77/322 (23.9%)	65/354 (18.4%)	0.016 ***
Hypertension (n)	40/92 (43.5%)	54/129 (41.9%)	96/205 (46.8%)	150/308 (48.7%)	161/307 (52.4%)	181/322 (56.2%)	194/359 (54.0%)	0.085 ***
Pulmonary disease (n)	3/92 (3.3%)	3/110 (2.7%)	11/205 (5.4%)	10/304 (3.3%)	20/306 (6.5%)	19/322 (5.9%)	25/323 (7.7%)	0.171 ***
Antibiotic type								
Group 1 (n)	**454 (90.8%)**	**313 (99.1%)**	**202 (59.9%)**	3 (0.7%)	7 (2.1%)	2 (0.6%)	2 (0.5%)	
Group 2 (n)	0 (0.0%)	0 (0.0%)	**130 (38.6%)**	**422 (98.1%)**	**218 (65.3%)**	**297 (91.1%)**	**390 (98.7%)**	
Group 3 (n)	1 (0.2%)	0 (0.0%)	0 (0.0%)	5 (1.2%)	**109 (32.6%)**	24 (7.4%)	3 (0.8%)	
Group 4 (n)	45 (9.0%)	3 (0.9%)	5 (1.5%)	0 (0.0%)	0 (0.0%)	3 (0.9%)	0 (0.0%)	
Cx type								
Cx1 (n)	10 (2.0%)	9 (2.8%)	11 (3.3%)	7 (1.6%)	4 (1.2%)	7 (2.1%)	8 (2.0%)	0.531 ***
Cx2 (n)	5 (1.0%)	8 (2.5%)	9 (2.7%)	5 (1.2%)	1 (0.3%)	0 (0.0%)	1 (0.3%)	0.031 ***
Cx3 (n)	4 (0.8%)	3 (0.9%)	7 (2.1%)	0 (0.0%)	0 (0.0%)	0 (0.0%)	1 (0.3%)	0.068 ***

* Analysis of variance, Tukey’s test. ** Kruskal–Wallis’ test, Tukey’s test. *** Pearson’s χ^2^ test or Fisher’s exact test, Tukey’s test. Groups accounting for 25% or more are displayed in bold. Group 1: patients who were prescribed oral quinolone as a prophylactic antibiotic for PB; Group 2: patients who were prescribed oral quinolone and intravenous aztreonam as prophylactic antibiotics for PB; Group 3: patients who were prescribed oral quinolones and intravenous aminoglycosides as prophylactic antibiotics for PB; Group 4: patients who were prescribed oral quinolones and intravenous quinolones as prophylactic antibiotics for PB.

**Table 3:** Baseline characteristics and complications of patients by year.

Period	1997–2007	2008–2009	2010–2011	2012–2013	2014–2015	2016–2017	2018–2019	*p* Value
Clinical cohort (n)	500	316	337	430	334	326	395	
Cx4 (n)	11 (2.2%)	9 (2.8%)	12 (3.6%)	6 (1.4%)	6 (1.8%)	9 (2.8%)	9 (2.3%)	0.495 *
Cx5 (n)	27 (5.4%)	14 (4.4%)	16 (4.7%)	17 (4.0%)	12 (3.6%)	9 (2.8%)	5 (1.3%)	0.049 *
HIRA cohort (n)				593	526	438	197	
Cx4 (n)				43 (7.3%)	31 (5.9%)	16 (3.7%)	3 (1.5%)	<0.01 *
Cx5 (n)				13 (2.2%)	11 (2.1%)	10 (2.3%)	5 (2.5%)	0.985 *
Antibiotic type								
Group 1 (n)				**232 (39.1%)**	**168 (31.9%)**	**116 (26.5%)**	46 (23.4%)	
Group 2 (n)				27 (4.6%)	20 (3.8%)	11 (2.5%)	4 (2.0%)	
Group 3 (n)				**306 (51.6%)**	**286 (54.4%)**	**265 (60.5%)**	**122 (61.9%)**	
Group 4 (n)				28 (4.7%)	52 (9.9%)	46 (10.5%)	25 (12.7%)	

* Pearson’s χ^2^ test or Fisher’s exact test, Tukey’s test. Groups accounting for 25% or more are displayed in bold. Group 1: patients who were prescribed oral quinolone as a prophylactic antibiotic for PB; Group 2: patients who were prescribed oral quinolone and intravenous aztreonam as prophylactic antibiotics for PB; Group 3: patients who were prescribed oral quinolones and intravenous aminoglycosides as prophylactic antibiotics for PB; Group 4: patients who were prescribed oral quinolones and intravenous quinolones as prophylactic antibiotics for PB. HIRA: Korea Health Insurance Review and Assessment Service.

**Table 4:** Univariate analysis of the clinical cohort according to the occurrence of SIRS after PB.

	Univariate Analysis	
	OR (95% CI)	*p* Value
Age	0.99 (0.94–1.05)	0.770
PSA	0.99 (0.91–1.00)	0.717
Prostate cancer	0.56 (0.15–1.64)	0.319
DM	2.22 (0.10–23.27)	0.515
Antibiotic type		
Group 1	Reference	
Group 2	0.11 (0.02–0.41)	0.004
Group 3	0.00	0.987
Group 4	1.47 (0.08–7.67)	0.713

Group 1: patients who were prescribed oral quinolone as a prophylactic antibiotic for PB; Group 2: patients who were prescribed oral quinolone and intravenous aztreonam as prophylactic antibiotics for PB; Group 3: patients who were prescribed oral quinolones and intravenous aminoglycosides as prophylactic antibiotics for PB; Group 4: patients who were prescribed oral quinolones and intravenous quinolones as prophylactic antibiotics for PB. OR, odds ratio; CI, confidence interval.

**Table 5:** Isolated bacteria and antibiotic susceptibility cultured in urine and blood from patients with SIRS.

	Group 1 (*n* = 983)	Group 2 (*n* = 1457)	Group 3 (*n* = 142)	Group 4 (*n* = 56)
Febrile UTI (n)	12	2	0	1
Urine culture				
*Escherichia coli*				
Quinolone R	0	0	0	0
ESBL	1 (2010)	1 (2019)	0	0
*Enterococcus faecium*	0	1 (2019)	0	0
*Citrobacter freundii*	1 (2010)	0	0	0
No growth	10	0	0	1
Blood culture				
*E. coli*				
Quinolone R	3 (2009 × 2, 2010)	0	0	0
ESBL	1 (2010)	1 (2019)	0	0
No growth	8	1	0	1

Group 1: patients who were prescribed oral quinolone as a prophylactic antibiotic for PB; Group 2: patients who were prescribed oral quinolone and intravenous aztreonam as prophylactic antibiotics for PB; Group 3: patients who were prescribed oral quinolones and intravenous aminoglycosides as prophylactic antibiotics for PB; Group 4: patients who were prescribed oral quinolones and intravenous quinolones as prophylactic antibiotics for PB. UTI, urinary tract infection; R, resistant; ESBL, extended-spectrum beta-lactamases.

## Data Availability

The data presented in this study are available on request from the corresponding author. The data are not publicly available due to restrictions imposed by the Gangneung Asan Hospital Institutional Review Board. In case of further questions, the telephone number of the person in charge of document access is +82-33-610-3018.
